# Vitamin D genetic risk scores in multiple sclerosis

**DOI:** 10.1007/s00415-022-11466-4

**Published:** 2022-11-05

**Authors:** Ashvin Kuri, Nicola Vickaryous, Amine Awad, Benjamin Meir Jacobs, Ruth Dobson

**Affiliations:** 1 Barts and The London School of Medicine and Dentistry, QMUL, London, UK; 2 Preventive Neurology Unit, Wolfson Institute of Population Health, Charterhouse Square, London, EC1M 6BW UK; 3 Department of Neurology, Royal London Hospital, London, UK; 4 Department of Neurology, Massachusetts General Hospital and Brigham and Women’s Hospital, Boston, MA USA

**Keywords:** Multiple sclerosis, Vitamin D, Genetic risk score

## Abstract

**Background:**

Low serum 25(OH)D_3_ (vD) is an environmental risk factor for multiple sclerosis (MS). Lower vD levels during early disease may be associated with long-term disability. Determinants of serum vD levels in healthy individuals include supplementation behaviour and genetic factors. These determinants have been less well studied in people with MS (pwMS).

**Methods:**

We developed a vD-weighted genetic risk score (GRS) and validated this in 373,357 UK Biobank participants without MS. We measured serum 25(OH)D_3_ and genotyped six vD-associated SNPs (rs12785878, rs10741657, rs17216707, rs10745742, rs8018720, rs2282679) in a cohort of pwMS (*n* = 315) with age and geographically matched controls (*n* = 232). We then assessed predictors of serum vD concentration in this cohort.

**Results:**

The GRS was strongly associated with vD status in the Biobank cohort (*p* < 2 × 10^–16^). vD supplementation, having MS, lower BMI, increased age and supplementation dose were associated with higher vD levels (false discovery rate, FDR < 5%). In multivariable models adjusting for supplementation, BMI, age, sex, and MS status, the GRS was strongly associated with vD level (*p* = 0.004), but not in those who supplemented (*p* = 0.47).

**Conclusions:**

Our findings suggest that vD supplementation is the major determinant of vD level in pwMS, with genetic determinants playing a far smaller role.

## Introduction

Low serum 25(OH)D_3_ (vD) is an environmental risk factor for multiple sclerosis (MS). Observational cohort and case–control studies have consistently shown that lower serum vD concentrations are associated with an increased risk of MS [1]. Mendelian randomisation studies have provided genetic evidence that low serum vD is likely to be a causal factor in MS development [2]. In addition, lower vD levels during early disease may be associated with long-term disease activity and progression [3]. Studies have shown that higher serum vD levels in people with MS are associated with a lower risk of clinical relapse [4] and slower rates of gadolinium-enhancing lesion accumulation on magnetic resonance imaging (MRI) [5]. However, it remains unclear whether vitamin D supplementation is able to modify disease activity in people with MS.

Serum vD is influenced by both a complex genetic architecture alongside environmental factors including sunlight exposure and supplementation [6, 7]. Studying the determinants of vD status in people with MS is potentially confounded as some determinants—such as latitude and variants in/near vD-associated genes (e.g. *CYP24A1*)—may themselves directly influence MS risk [6–8].

In this study, we evaluated the determinants of serum vD level in a cohort of people with MS and matched controls. We collected retrospective self-reported survey data on environmental factors associated with vD and genotyped the cohort at six vD-associated SNPs to construct a 6-SNP genetic risk score (GRS). We measured vD serum levels using dried blood spots and evaluated the relationship between genetic factors, environmental factors, and vD in both the MS and control cohorts.

## Methods

### Study Recruitment

The study involved two separate cohorts. The first cohort, containing only people without MS, evaluated the vD GRS and consisted of 354,475 healthy European-ancestry UK Biobank participants [9].

**Fig. 1:**
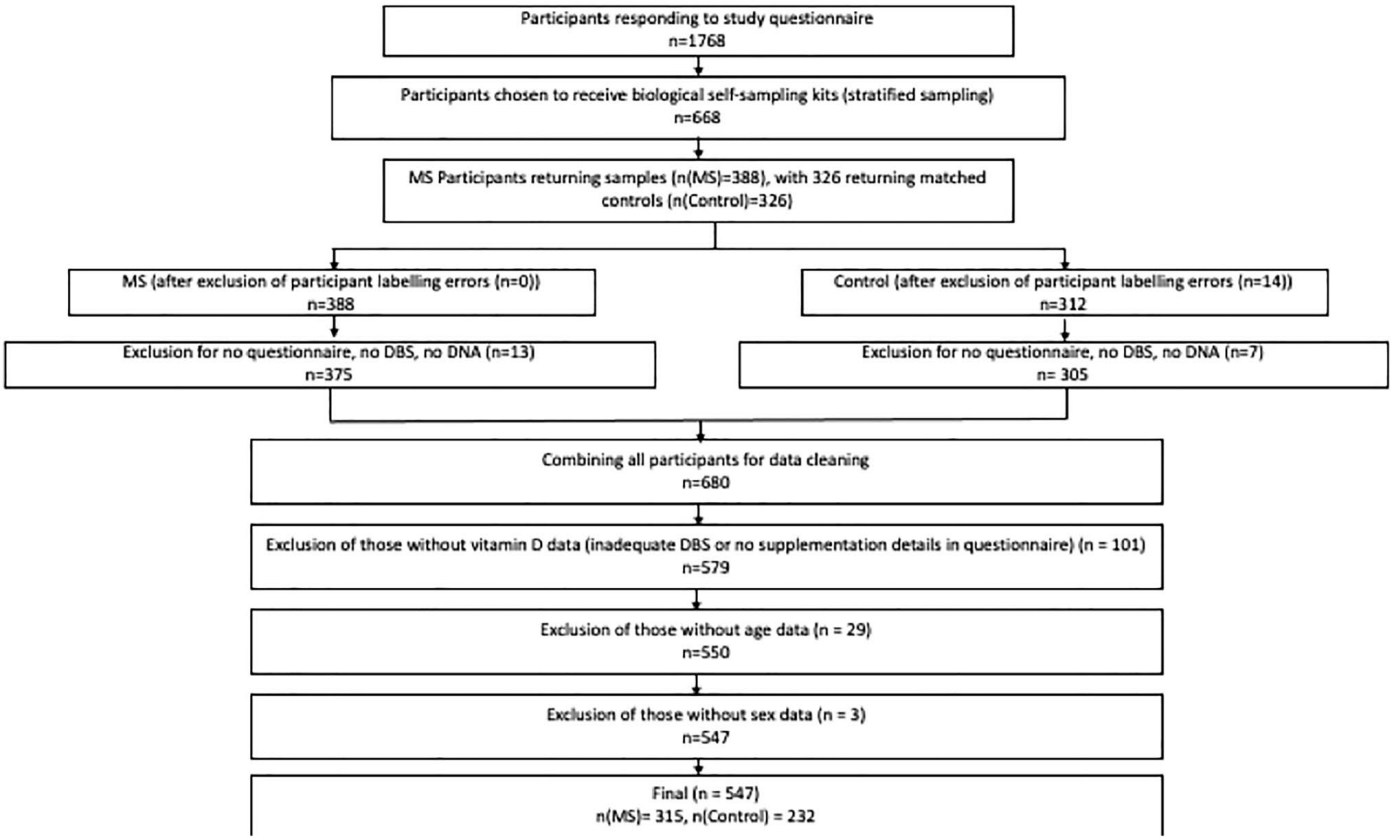
Selection of participants for the study cohort

The second (study cohort) was a cohort of MS participants and matched controls, as described in our previous work [10]. In brief, in this study cohort, people with MS were recruited through the UK MS Register and regional MS networks. Each enrolled person with MS was asked to recruit an unrelated friend of the same gender, age (within 5 years), and geographical location (within a 50-mile radius) to act as a matched control. Figure 1 details a flow chart of the study cohort recruitment and resulting study population, highlighting sources of missing data. Sources of missing data included non-return of any biological sampling, inadequate (low quality/unmeasurable) biological samples, or no/inadequate response to demographics or supplementation questions within the questionnaire. Importantly, as described in our previous paper, we used stratified random sampling of our initially recruited population to ensure balance across geographical areas, MS disease types, and EDSS in the final study population [10]. This was achieved through random sampling within participant groups that were stratified based on geographical location (100 km × 100 km square), MS type (RRMS, SPMS, PPMS) and disability (EDSS < 6 or EDSS > 6). People with MS undergoing contemporaneous steroid treatment (within 2 months prior to enrolment) were excluded from recruitment. Our final study cohort population consisted of 315 people with MS and 232 controls.

### Ethics

This study had ethical permission via London Stanmore REC (18/LO/1455). UK Biobank has separate ethical approval; this work was performed under application 43,101 (REC approval 11/NW/0382). All participants gave informed consent on Biobank registration and are free to withdraw from the study at any point, at which point their data are censored and cannot be included in further analyses.

### Biological sampling, 25(OH) vitamin D analysis and SNP genotyping

As previously described, all participants within the study cohort were sent a remote sampling kit [10]. Each sampling kit contained a dried blood spot sampling system (serum vD analysis), buccal swab (genetic material) and questionnaire (demographics). Sampling packs were sent out between February and July 2019. Importantly, our previous work analysing this cohort showed the influence of ‘season of sampling’ on vitamin D level was non-significant within this cohort [10]. Serum vD levels were determined from dried blood spots, using a previously described protocol involving liquid chromatography-tandem mass spectrometry [10]. Two DBS were analysed per participant; results were excluded if duplicate analysis differed by ≥ 15%, if only one viable DBS was available, or if DBS were deemed to be of poor quality, i.e., spots too small, not fully soaked through, or multiple overlapping spots. Questionnaires collected a range of demographic and MS-specific data including geographical location, gender, age, BMI, smoking status, MS type, EDSS, MSIS, date of diagnosis and supplementation use, frequency, and dose.

The six genotyped SNPs used to construct the GRS were selected from the first-stage discovery meta-GWAS in the SUNLIGHT study (Table 1) [7]. Effect sizes summarised in Table 1 were those calculated in SUNLIGHT [7]. In the UK Biobank cohort, rs6538691 was used as a proxy SNP for rs10745742 (*R*^2^ > 0.99), with the T alleles corresponding. In the study cohort the SNP rs2282679, in complete linkage disequilibrium with rs3755967, was used as a proxy (*R*^2^ = 0.9719, correlated alleles: C = T, T = G). Genotyping and QC protocols for UK Biobank are described in detail elsewhere [11]. Taqman SNP genotyping (Thermofisher Scientific) was used for the MS study cohort, for 5/6 SNPs (rs12785878, rs10741657, rs17216707, rs8018720, rs2282679), on a QuantStudio 5 Real-Time PCR system (*Applied Biosystems*) using the manufacturer’s recommended protocol, and analysed with TaqMan Genotyper Software (*Thermofisher Scientific*). For 1 SNP (rs10745742), genotyping was performed by pyrosequencing on a PyroMark Q48 Autoprep (*Qiagen*), followed by manual QC with a 97.5% call rate.

**Table 1:** SNPs and their respective effect sizes used in the 6-SNP GRS in this study

SNP	Effect/reference Alleles (effect allele in bold)	Effect size of effect allele (*β* = per-allele unit increase in vitamin D Z-score)
AMDHD1 (rs10745742)	**T**/C	0.017
DHCR7 (rs12785878)	**T**/G	0.036
CYP2R1 (rs10741657)	**A**/G	0.031
CYP24A1 (rs17216707)	**T**/C	0.026
SEC23A (rs8018720)	**C**/G	− 0.017
GC (rs2282679) (proxy SNP for rs3755967)	**T**/C	− 0.089

### Genetic risk score

Prior to GRS calculation in the UK Biobank population, we performed standard individual-level and variant-level quality control, removing individuals with high missingness (> 10%), non-European genetic ancestry, and variants with a low MAF (< 0.01), extreme deviation from hardy–weinberg (*P* < 1*e *− 5), high genotype missingness (> 10%), or low imputation score (*R*^2^ < 0.3). All 6 SNPs passed quality control in UKB.

Individual GRSs were calculated using the formula:

$$\mathrm{GRS}={\sum }_{j=1}^{m}{Xj}\beta j,$$

where *m* represents the number of included SNPs (*m* = 6), *X* represents the copy number of a given risk-increasing allele and *β* represents the GWAS-generated beta value (per allele unit increase in Z-score). This generated a 6-SNP GRS, with risk-increasing alleles weighted by their weights (*j*). Where *β* was negative (rs2282679, rs8018720), the non-effect allele copy number was used instead to maintain positive effect sizes.

### Statistical analysis

Data were analysed in R (v4.0.0) using RStudio (v1.2.5042). Genotype distribution differences were evaluated using Chi-square tests. Predictors of Z-score normalised serum vD levels were assessed with linear regression. Associations between GRS and serum vD level were assessed using multivariable linear regression models adjusting for confounding variables. All codes used in this study are available on GitHub (https://github.com/benjacobs123456/VitaminD_UKMSRegister).

## Results

### Baseline characteristics

Demographics for both the UK Biobank and study cohort were evaluated separately. Within the cohort containing only people without MS, and recruited from UK Biobank (median age 58.0 [IQR 12], 53.3% female, 100% White European ethnic background, 84.4% born in England, Townsend deprivation index Z scores median − 2.35 [3.8]), 5.1% (18,882/373,357) of the cohort reported supplementing, but dose information was not available.

In the study cohort (MS: 74% female, median age 54.0; Control: 75% female, median age 56.0), a higher proportion of MS participants took supplementation compared to controls (71.1% vs 26.7%) and among all supplementers, doses tended to be higher in the MS cohort (MS median dose: 1200 IU/day vs control median dose: 571 IU/day).

### Association between GRS and serum vitamin D level

We first tested the association of the 6-SNP GRS with serum vitamin D status among non-supplementers of European ancestry in UK Biobank. In this cohort (*n* = 354,475) the GRS was strongly associated with vD status (0.14 SD increase in vD per 1 SD increase in GRS Z-score, *p* < 2 × 10^–16^). This estimate was similar among the subset of supplementers (*n* = 18,882, 0.17 SD increase in vD per 1 SD increase in GSR Z-score, *p* < 2 × 10^–16^).

We then evaluated the predictors of vD levels in our study cohort using regression models adjusted for age and sex. We found that supplementation, supplementation dose, BMI and MS status were the best predictors of serum vD levels (false discovery rate: < 5%). The association of MS status with serum vD level within this model was explained by the larger median supplementation dose in people with MS (median dose: 1200 IU/day) compared with matched controls (median dose: 571 IU/day).

**Fig. 2:**
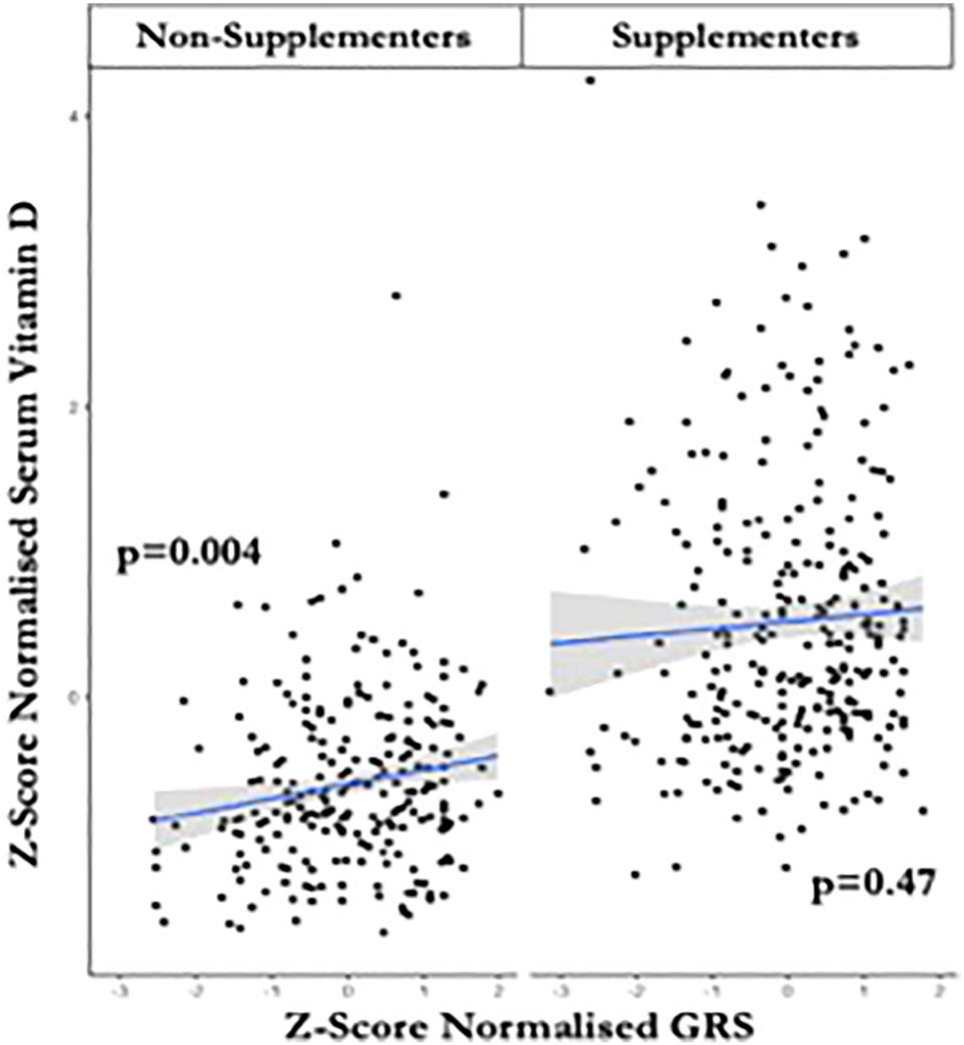
GRS is significantly associated with vitamin D level in non-supplementers but is not significantly associated with vitamin D level in supplementers. The association of GRS with vitamin D level in the study cohort (pwMS + matched controls) was evaluated through multivariable models, adjusting for supplementation, BMI, age, sex and MS status. The graph represents the regression of Z-Score normalised serum vitamin D on Z-score normalised GRS in non-supplementers (left) and supplementers (right), after adjusting for BMI, age, sex and MS status

In multivariable models adjusting for age, sex, BMI, supplementation, and MS case/control status, the GRS was associated with a 0.1-SD increase in serum vD (*p* = 0.004). However, there was no evidence for a relationship between GRS and serum vD in those who supplemented (*p* = 0.47) (Fig. 2). Addition of the GRS to a model including age, sex, BMI, supplementation, and MS case/control status only led to a minor improvement in model fit, increasing the adjusted *R*^2^ from 0.37 to 0.39, i.e., equating to an extra 2% of the variation in vD levels explained.

## Discussion

Our findings suggest that supplementation is an important determinant of serum vD levels in people with MS. Our GRS performed well in the UK Biobank cohort and showed significant association with serum vD level in non-supplementers in our study cohort. The loss of association of GRS with serum vD level in those that supplement suggests a greater influence of supplementation compared with vD-associated genetic variants.

It is important to recognise the limitations of this study. The limited overall heritability of vitamin D levels (~ 7.5% in SUNLIGHT) places an upper bound on the performance of the GRS, and the variants used in this study are expected to explain ~ 2.4% of the overall variance in vitamin D status. Another limitation of the study was our method of capturing serum vD level and participant-reported supplementation dose. In our study, participants only returned DBS for serum vD concentration evaluation at a single timepoint and reported their supplementation dose at a single timepoint. There is considerable evidence for seasonal fluctuation in serum vD concentration and thus serial measurements at different timepoints to capture temporal variations may have improved the study design [12]. Additionally, participants may change their supplementation habits according to seasonal changes (such as increasing supplementation during the winter months), and this was also not captured by our data collection method. However, our analysis did not demonstrate such seasonal variation, likely due to supplementation throughout the calendar year.

We also recognise that our control cohort within the study population had a higher proportion of supplementers (26.7%) compared to the UK Biobank population (5.1%). A potential explanation for this finding is our recruitment method of participant-recruited ‘matched controls.’ This may have resulted in a cohort that is more attuned to the benefits of vitamin D supplementation; additionally, participants in this study knew that they were taking part in a vitamin D-related study, which is likely to have introduced some bias. We would also suggest that as UK Biobank data is only collected once, at baseline, supplementation habits may have changed contemporaneously over time, which may also explain this finding. A final limitation of the study is the relatively homogeneous ethnicity of the cohort—as vitamin D levels are influenced by skin tone, and MS is increasingly common in individuals who identify as Black British and Asian in the UK, this is an important area of further study [13].

In conclusion, our study provides evidence that supplementation is an important determinant of vD status in people with MS, and likely trumps the relatively minor effects of common genetic variation in determining vD status. Future work should focus on validating these findings in larger cohorts, constructing more complete GRSs and further characterising the role of vD in MS aetiology and pathogenesis from a functional and epidemiological perspective.

## Data Availability

UK Biobank (UKB) data is available to researchers on application directly to the UK Biobank team (details available: https://www.ukbiobank.ac.uk/enable-your-research/apply-for-access). All data pertaining to people with MS within our cohort is available within the UK MS Register via application (details available: https://ukmsregister.org/Research/WorkingWithUs).
